# Permeability of dopamine D2 receptor agonist hordenine across the intestinal and blood-brain barrier *in vitro*

**DOI:** 10.1371/journal.pone.0269486

**Published:** 2022-06-16

**Authors:** Maria Hahn, Viktoria Lindemann, Matthias Behrens, Dennis Mulac, Klaus Langer, Melanie Esselen, Hans-Ulrich Humpf

**Affiliations:** 1 Institute of Food Chemistry, Westfälische Wilhelms-Universität Münster, Münster, Germany; 2 Institute of Pharmaceutical Technology and Biopharmacy, Westfälische Wilhelms-Universität Münster, Münster, Germany; Lewis Katz School of Medicine, Temple University, UNITED STATES

## Abstract

Hordenine, a bioactive food compound, has several pharmacological properties and has recently been identified as a dopamine D2 receptor (D2R) agonist. Since the pharmacokinetic profile of hordenine has been described to a limited extent, the present study focused on the transfer and transport of hordenine across the intestinal epithelium and the blood-brain barrier (BBB) *in vitro*. Hordenine was quickly transferred through the Caco-2 monolayer in only a few hours, indicating a rapid oral uptake. However, the high bioavailability may be reduced by the observed efflux transport of hordenine from the bloodstream back into the intestinal lumen and by first pass metabolism in intestinal epithelial cells. To determine the biotransformation rate of hordenine, the metabolite hordenine sulfate was synthesized as reference standard for analytical purposes. In addition, transfer studies using primary porcine brain capillary endothelial cells (PBCEC) showed that hordenine is able to rapidly penetrate the BBB and potentially accumulate in the brain. Thus, a D2R interaction of hordenine and activation of dopaminergic signaling is conceivable, assuming that the intestinal barrier can be circumvented by a route of administration alternative to oral uptake.

## Introduction

**Fig 1:**
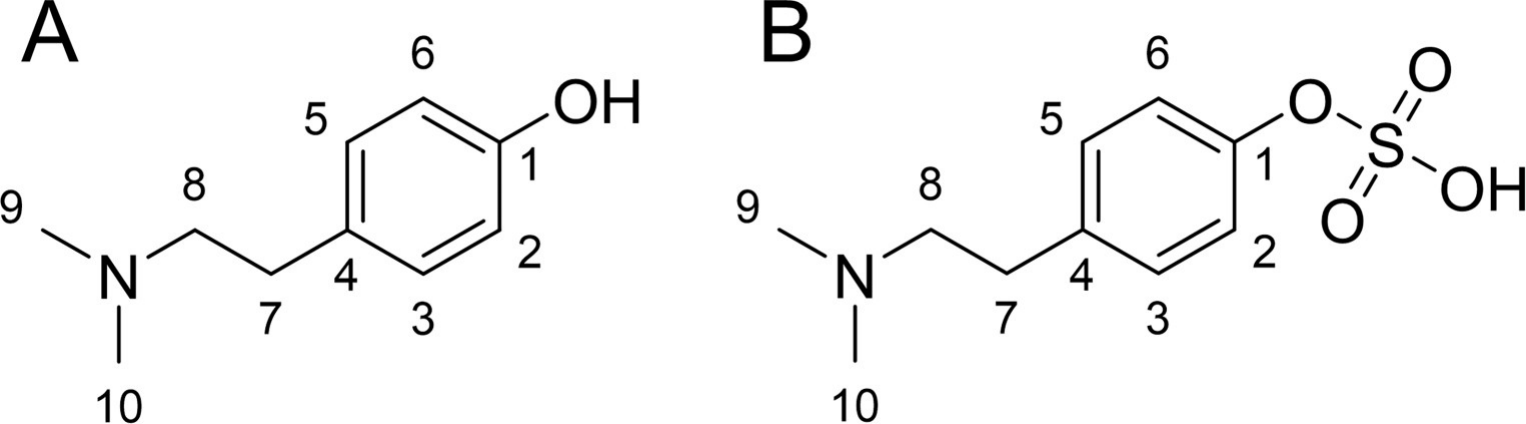
Chemical structures of hordenine (A) and its metabolite hordenine sulfate (B).

Hordenine (Fig 1A), a phenylethylamine alkaloid, has been recently associated with several bioactive and pharmacological properties including anti-inflammatory, anti-diabetic and anti-fibrotic effects [1]. The alkaloid occurs naturally in various plants, including grasses [2], cacti [3] and bitter oranges [4]. One of the main sources of hordenine is germinated barley (*Hordeum vulgare* l.), which can release hordenine into beer during the brewing process. Several types of beer contain hordenine concentrations ranging from 1.0 to 6.3 mg/L, suggesting that beer consumption may contribute to a large proportion of dietary hordenine intake [5].

Hordenine was recently identified as a food-derived dopamine receptor D2 (D2R) agonist *in vitro*, with receptor activation appearing to be more sustained than activation by the endogenous neurotransmitter dopamine [6]. Signal transduction through this receptor controls physiological functions related to locomotion, hormone production and drug abuse [7]. Moreover, D2R and their regulatory molecules are known therapeutic targets, whereas dysregulation of dopaminergic signaling is associated with neurological and psychiatric diseases such as Parkinson’s disease (PD), schizophrenia, and depression [8]. PD is characterized primarily by degeneration of dopaminergic neurons in *substantia nigra*. The most effective therapeutic approach for compensating the dopamine deficiency remains levodopa, a precursor of dopamine. Levodopa, in contrast to dopamine, can overcome the blood-brain barrier (BBB) and is metabolized in the brain to the biologically active dopamine [9]. Treatment of PD with levodopa results in significant relief of motor disorders, however, efficacy often wanes, and adverse effects appear with long-term therapy. Therefore, dopamine receptor agonists have been used for several years to delay the initiation of levodopa therapy or as adjunct therapy together with levodopa. Despite their efficacy, the dopamine receptor agonists may cause critical side effects such as fibrotic valvular heart disease and dopamine dysregulation syndrome [9, 10].

D2R dysfunctions are associated with neurological and psychiatric disorders on the one hand, and food reward and addictive behavior on the other. Dietary food intake of palatable food leads to dopamine release in the brain, resulting in activation of dopaminergic pathways and interaction with D2R [11]. To determine whether the hordenine levels of beer are sufficient to interact with the D2R, a pilot study with four volunteers was conducted to investigate the biokinetics of hordenine after oral beer consumption. Maximum plasma levels of free hordenine were 12.0–17.3 nM after 0–60 min. Previous studies showed an EC_50_ value for hordenine at D2R of 3.7 μM and an inhibitory constant *K*_*i*_ of 13 μM. Thus, beer consumption and the resulting plasma concentrations of hordenine in the low nanomolar range might not lead to direct D2R effects of hordenine [6, 12]. However, higher hordenine concentrations than natural food and beverage consumption could result from the intake of highly concentrated dietary supplements. Therefore, the pharmacokinetics of hordenine were investigated after administration of a hordenine-rich dietary supplement in three healthy volunteers. The highest concentration of hordenine in human plasma was observed after 65 ± 14 min with 16.4 ± 7.8 μg/L (≈ 99 ± 47 nM). Although, oral administration of 100 mg hordenine from a dietary supplement causes higher uptake compared to regular nutrition, the resulting plasma hordenine levels remain lower than the published EC_50_ and *K*_*i*_ of D2R interaction. This dose is therefore unlikely to induce a direct interaction with D2R [13]. The agonistic activity of hordenine towards D2R has already been confirmed [6]. However, the important question whether hordenine can reach the CNS to interact with dopamine receptors has only been marginally studied. In addition, the bioavailability of hordenine and its metabolic fate have not been clearly elucidated, so far. In the preliminary biokinetic studies, hordenine and the two phase-II metabolites, hordenine sulfate and hordenine glucuronide, were observed in human plasma immediately after oral administration, indicating a rapid absorption and biotransformation of hordenine [12, 13]. Nevertheless, the pharmacokinetics and pharmacodynamics of hordenine require further investigation.

Thus, the first aim of the present study was to investigate the intestinal permeability of hordenine using the Caco-2 model. Human Caco-2 cells are capable of spontaneous differentiation into cell monolayers with morphological and functional characteristics of enterocytes in the small intestine [14, 15]. Caco-2 cells were cultivated on membrane filters to study the transfer rate of hordenine through the small intestinal epithelium and to assess whether hordenine is bioavailable in the systemic circulation. In addition, we synthetized an analytical standard of hordenine sulfate (Fig 1B) and developed a high-performance liquid chromatography-tandem mass spectrometry (HPLC-MS/MS) method for the simultaneous quantitation of hordenine and its metabolite hordenine sulfate in cell culture samples.

In the second part of the study, the transfer properties of hordenine across the blood-brain barrier (BBB) were analyzed. In general, the BBB consists of astrocytes, pericytes and endothelial cells, which form a neurovascular unit regulating the flux of endogenous and exogenous compounds from the bloodstream to the brain or *vice versa* [16]. The used *in vitro* BBB model is based on the cultivation of primary porcine brain capillary endothelial cells (PBCEC) on membrane filter inserts. The overall objective of the present study was to further elucidate the biokinetics of hordenine and to obtain an initial assessment of its permeation trough the BBB and to enable D2R interactions.

## Materials and methods

### Chemicals and reagents

All solvents and chemicals used in this study were purchased at Fisher Scientific (Schwerte, Germany), Carl Roth (Karlsruhe, Germany), VWR (Darmstadt, Germany) or Sigma-Aldrich (Steinheim, Germany). Water was purified with a Purelab flex 2 system (ELGA LabWater Veolia Water Technologies, Celle, Germany). Cell culture media and supplements were obtained from PAN-Biotech GmbH (Aidenbach, Germany), Merck (Darmstadt, Germany) and Thermo Fisher Scientific (Dreieich, Germany). Hordenine with a purity ≥ 97% was purchased from Sigma-Aldrich (Steinheim, Germany).

Rat tail collagen was isolated according to a previous protocol by Franke and co-workers [17] with some modifications. After cleaning and sterilizing the rat tails in 70% (*v*/*v*) ethanol for 30 min, the rat tails were clamped at the tail end with hemostatic forceps, bent, and the collagen fibers were obtained by slowly pulling them out. The collagen fibers were then incubated with sterile 0.1% acetic acid for 24 h at 4°C to dissolve them. The suspension was centrifugated at 5000 rpm for 30 min, and the supernatant was collected. The pellet was mixed with 0.1% acetic acid, centrifugated, and the supernatants combined. Finally, the protein concentration of the solution was determined.

### Viability assay in Caco-2 cells

For the following studies, the human colon adenocarcinoma (Caco-2) cells from American Type Culture Collection (ATCC, Manassas, Virginia, USA) were used. The protocols for cultivation of Caco-2 cells and viability testing are described in detail in our previous publication [18]. To ensure that the applied hordenine concentration causes no cytotoxic effects, Caco-2 cells were incubated with 0.1 μM to 100 μM hordenine (by dilution of 100 mM stock solution in DMSO) for 48 h. Cellular viability was determined using the resazurin reduction assay based on O’Brien [19].

### Viability assay in PBCEC

For evaluation of cellular viability of PBCEC, the Cell Counting Kit-8 (CCK-8) from Dojindo Laboratories (Tokyo, Japan) was used based on in-house experiments. Isolation and preparation of PBCEC were performed by the group of Prof. Dr. Langer, Institute of Pharmaceutical Technology, WWU Münster. The cultivation procedure of PBCEC is based on earlier protocols [17, 20]. Briefly, cryopreserved PBCEC were rapidly thawed at 37°C, resuspended in serum-containing medium (Medium 199 Earle’s with 10% fetal calf serum, 4.1 mM l-glutamine, 100 U/mL penicillin, 100 μg/mL streptomycin, 100 μg/mL gentamycin) and centrifugated at 220 × *g* at 20°C for 10 min. Afterwards, the supernatant was removed, fresh medium was added, and the desired cell density was prepared by dilution. 1.5 × 10^4^ cells per well (100 μL) were seeded on rat tail collagen coated 96-well plates and were cultivated at 37°C, 5% CO_2_ under saturated humidified conditions for 48 h. To induce cell differentiation and endothelial phenotype, the medium was replaced with serum-free medium (DMEM/Ham’s F-12 1:1 with 0.7 mM l-glutamine,100 U/mL penicillin, 100 μg/mL streptomycin, 100 μg/mL gentamycin) supplemented with 550 nM hydrocortisone (Sigma-Aldrich, Steinheim, Germany). 96 h after seeding, cells were treated with various hordenine concentrations (0.1 μM-100 μM in serum-free medium) over 48 h following the addition of 10 μL WST-8 (2-(2-methoxy-4-nitrophenyl)-3-(4-nitrophenyl)-5-(2,4-disulfophenyl)-2H-tetrazolium, monosodium salt) solution and incubation for 70 min at 37°C, 5% CO_2_ in a saturated humidified atmosphere. The reduction of the tetrazolium salt to a formazan dye based on the active dehydrogenase of viable cells and the formazan absorbance was analyzed at *λ* = 457 nm with *λ* = 650 nm as a reference using an Infinite M200 PRO microplate reader with Tecan i-control software version 1.7.1.12 (Tecan, Crailsheim, Germany). After subtraction of the blank absorbance, the determined viability of the cells incubated with hordenine was normalized to a solvent-adjusted negative control.

### Passive transfer studies

#### Caco-2 model

For intestinal transfer studies, cryopreserved Caco-2 cells were freshly thawed, cultivated in serum-containing DMEM/F-12 cultivation medium for 7-9 days, and seeded on 12-well Transwell^®^ filter inserts with microporous polycarbonate membranes (1.12 cm^2^ growth area, 0.4 μm pore size; Corning, Wiesbaden, Germany). The transfer studies were performed following our previous study [18]. The only difference was the use of a different model of the cellZscope^®^ system (nanoAnalytics, Münster, Germany) with different volumes of the filter inserts. The apical compartment was 760 μL and the basolateral compartment was 1650 μL (ratio 1:2.17). For substance incubation, this resulted in the exchange of 76 μL of HBSS in the apical compartment with 76 μL of a 10 μM hordenine solution. Therefore, the final concentration of hordenine was 1 μM. In the transfer studies, 46 μL samples were taken from the apical compartment and 100 μL from the basolateral compartment. The obtained samples were analyzed and quantified using HPLC-MS/MS. Permeability coefficients were calculated according to equation 1 und equation 2 in the S1 Equation of S1 File.

#### BBB model

The model system for the BBB was based on the same principles as the Caco-2 monolayer system. The main difference was the distribution of the compartments. The apical compartment represented the “blood-side”, and the basolateral compartment reflected the “brain-side”. Based on the thawing and seeding procedure as described for the viability assay, PBCEC (2.5 × 10^5^ cells in 500 μL serum-containing PBCEC cultivation medium per filter) were seeded onto rat-tail collagen-coated Transwell^®^ filter membranes (1.12 cm^2^ growth area, 0.4 μm pore size) in the apical compartment. After a cultivation period of 48 h, the medium was replaced with serum-free medium supplemented with hydrocortisone. Two days of differentiation phase followed and hordenine (final concentration: 1 μM in serum-free medium) was incubated at 37°C, 5% CO_2_ in a saturated humidified atmosphere. The application of hordenine, the analysis of the barrier integrity and the sampling procedure were performed according to the Caco-2 model section. To analyze the transfer rates of hordenine across the barrier, samples were collected from both compartments after eight points in time (0.5, 1, 2.5, 6.5, 18, 24, 42 and 48 h) and quantified by HPLC-MS/MS. Permeability coefficients were calculated according to equation 1 und equation 2 in the S1 Equation of S1 File.

#### Barrier integrity

For monitoring tight junction and cell monolayer integrity, transepithelial/transendothelial electrical resistance (TEER) and electrical capacitance (*c*_CL_) of the cell monolayers were analyzed using the cellZscope^®^ cellular impedance spectroscope (nanoAnalytics, Münster, Germany). The integrity of the barriers was confirmed by application of 50 μM Lucifer Yellow (LY) in the apical compartment. After 1 h, the concentration of LY in the basolateral compartment was determined by fluorescence analysis at *λ*_ex_ = 430 nm and *λ*_em_ = 540 nm using the same microplate reader as for the cytotoxicity assay.

### Active transport studies

Active transport studies were carried out by applying equimolar concentrations of 200 nM hordenine in both compartments of the cell monolayers. Cultivation, handling and incubation of the Caco-2 cells and PBCEC, as well as sampling for 48 h and quantitation of hordenine followed the same scheme as for the passive transport studies.

### Synthesis of hordenine sulfate

The synthesis of hordenine sulfate was achieved following the protocols for the sulfation of *p*-hydroxymethamphetamine [21] and sulfation of flavonoids [22] with some modifications. 81 mg of hordenine were dried overnight with 401 mg of a pyridine-sulfur trioxide complex. After flushing three times with argon, 4 mL pyridine was added to the reaction mixture, and the suspension was stirred for 24 h at 40°C under argon atmosphere. After transferring the reaction mixture to a glass centrifuge tube, the suspension was washed three times with 5-10 mL pyridine and centrifuged at 475 x *g* and room temperature each time. Subsequently, the supernatants were combined, slightly alkalized with aqueous 0.1 M sodium hydroxide solution and evaporated to dryness. The precipitate of the reaction mixture was alkalized as well. Precipitate and supernatant were reconstituted in water and analyzed for the reaction products by HPLC-high resolution mass spectrometric (HRMS) analysis (see HPLC-HRMS analysis of hordenine sulfate).

#### Purification of hordenine sulfate

For separation in hydrophilic interaction liquid chromatography (HILIC) mode using the TSKgel^®^ amide-80 column, the samples were diluted with acetonitrile (ACN) to match the initial eluent composition of ACN/water (87+13, *v/v*). Purification of hordenine sulfate was performed by semipreparative Jasco HPLC PU-2087/2087 system and UV-2075 detection (Jasco, Groß-Umstadt, Germany). For separation, a 300 × 7.8 mm TSKgel^®^ amide-80 column (10 μm) (Tosoh Bioscience, Griesheim, Germany) was used. The column oven temperature was set to 40°C. A binary gradient consisting of ACN/water (87+13, *v/v*) (eluent A) and water (eluent B), both containing 0.01% formic acid (FA), and a flow rate of 2 mL/min was applied. The solvent composition resulted from optimization of a previous isocratic method. The gradient started with 100% A, which was held for 12 min. After a fast decrease to A to 50% at 12.1 min, A was held for 9 min, followed by an increase to 100% of A for further 9 min to equilibrate the column for the next injection. The wavelength of the UV detector was set to 266 nm. The collected fractions were immediately alkalized with aqueous 1 M sodium hydroxide solution at a ratio of about 1/500 of the eluent volume to inhibit sulfate hydrolysis. Fractions containing hordenine sulfate were individually combined. Subsequently, the solvent was removed on a rotary evaporator and the product was freeze-dried. The purity of the synthesis product was determined based on HPLC with evaporative light-scattering detection as described in S1 Table of S1 File. Hordenine sulfate was obtained as a white powder with a yield of 110.9 mg (92%) and purity of 99%.

#### HPLC-HRMS analysis of hordenine sulfate

The HPLC-HRMS analysis was performed using a Bruker Daltonics GmbH & Co. KG (Bremen, Germany) Elute HPLC system coupled with a Bruker impact II qTOF mass spectrometer. Chromatographic separation of the substances was achieved using a 150 × 2 mm Nucleodur π^2^ column (3 μm) equipped with a 4 × 2 mm Nucleodur π^2^ precolumn (Macherey-Nagel, Düren, Germany). Further HPLC parameters are described in HPLC-MS/MS settings (for method see Quantitation of hordenine and hordenine sulfate by HPLC-MS/MS). The qTOF mass spectrometer was equipped with an ESI Apollo II ion source operated in negative ionization mode. The following parameters were applied for HRMS acquisition: capillary temperature: 250°C, dry gas: 12 L/min, nebulizer gas: 4 bar, capillary voltage: -3 kV. For identification of metabolites, a full scan mode within a mass range of *m*/*z* 50 to 850 as well as Auto MS/MS scan mode with spectra rate of 2 Hz were used. The collision energy was calculated from *m*/*z* 100 = 40 eV, *m*/*z* 500 = 60 eV, *m*/*z* 1000 = 80 eV. The obtained HRMS data of synthesized hordenine sulfate are shown in S1 Dataset.

#### NMR analysis

For structural elucidation, one- and two-dimensional nuclear magnetic resonance (NMR) experiments were performed on an Agilent DD2 600 MHz spectrometer (Agilent, Waldbronn, Germany). Therefore, the synthesized compound was dissolved in deuterated dimethyl sulfoxide (DMSO‑*d*_6_). Data were processed using the MestReNova software (12.0.4–22023, Mestrelab Research S.L., Santiago de Compostela, Spain) and Excel 2019 software (Microsoft Corporation, Redmond, USA), respectively. The recorded NMR spectra of hordenine sulfate are displayed in S1– S4 Figs of S1 File).

Quantitative ^1^H-NMR (qNMR) spectrum (S5 Fig in S1 File) was recorded to calculate the concentration of a hordenine sulfate solution, whereas deuterated dimethyl sulfoxide (DMSO‑d_6_) was used as solvent and thymol (Sigma Aldrich, Taufkirchen, Germany) as internal standard for quantitation. Quantitative determination was based on modification of an already existing method [23].

### Quantitation of hordenine and hordenine sulfate by HPLC-MS/MS

For the quantification of hordenine and hordenine sulfate by HPLC-MS/MS, a 1200 series HPLC system (Agilent, Waldbronn, Germany) coupled to an API 3200 mass spectrometer (SCIEX, Darmstadt, Germany) was used. Both devices were operated with Analyst Version 1.6.2 software (SCIEX, Darmstadt, Germany). Chromatographic separation of the substances was achieved using a 150 × 2 mm Nucleodur π^2^ column (3 μm) equipped with a 4 × 2 mm Nucleodur π^2^ precolumn (Macherey-Nagel, Düren, Germany). Hordenine and hordenine sulfate were separated using a binary gradient consisting of methanol (solvent A) and water (solvent B) both supplemented with 0.1% FA. After constant initial conditions of 5% A until 1 min, A was increased to 95% within 1 min and held for 2 min. Subsequently, the initial conditions were reapplied at 5 min for additional 5 min to re-equilibrate the column. The first 1.5 min and last 3 min of each run were discarded using a diverter valve to remove salts and polar cell culture components. The column oven temperature was operated at 45°C, and the flow rate was set to 400 μL/min. An injection volume of 10 μL was used for samples obtained in the transport studies.

The concentration of hordenine and hordenine sulfate was determined directly in cell culture medium using a matrix matched calibration. The incubation solution of hordenine applied in the transport studies and the synthesized standard of hordenine sulfate were used to generate a calibration curve from pre-incubated medium. The serum-free medium and HBSS buffer were previously incubated for 48 h with the respective cells and approximately matched the matrix of the analyzed samples. Thus, the use of an internal standard was not necessary.

Electrospray ionization (ESI) was applied in positive ionization mode with a spray voltage set to 5500 V and a source temperature of 450°C. The gas parameters were set to: curtain gas, 1.03 × 10^5^ Pa (15 psi), nebulizer gas, 2.41 × 10^5^ Pa (35 psi), and heater gas, 3.10 × 10^5^ Pa (45 psi). The collision gas was 6.89 × 10^4^ Pa (10 psi). The declustering potential (DP), the collision energy (CE) and the collision cell exit potential (CXP) were optimized for hordenine and hordenine sulfate by injection of a standard solution into the mass spectrometer using a syringe pump. The MS was operated in the multiple reaction monitoring (MRM) mode with each transition reaction monitored for 100 ms (dwell time). The following parameters were determined for the optimized MRM transitions: hordenine, DP = 25 V, CXP = 4 V, EP = 10 V, *m*/*z* 166 → 121 (CE = 22 V), *m*/*z* 166 → 103 (CE = 37 V), *m*/*z* 166 → 77 (CE = 50 V); hordenine sulfate, DP = 40 V, CXP = 6 V, *m*/*z* 246 → 166 (CE = 19 V, EP = 6 V), *m*/*z* 246 → 121 (CE = 35 V, EP = 7 V); *m*/*z* 246 → 77 (CE = 72 V, EP = 5 V).

### Statistical analysis

Cellular viability studies were performed in three independent experiments with six replicates (*n* = 3 × 6). Passive transport studies including TEER and *c*_CL_ analysis were performed in three independent experiments, each with three replicates (*n* = 3 × 3). For active transport studies, three independent cell preparations/passages with three replicates were used. (*n* = 3 × 3). Data are presented as mean ± standard deviation. All data were statistically evaluated using Excel 2013 (Microsoft Corporation, Redmond, USA) and an unpaired heteroscedastic Student’s *t*-test. Significant differences between data sets were marked with * (low significant, *p* < 0.05); ** (medium significant, *p* < 0.01); *** (highly significant, *p* < 0.001).

## Results

In the present study, the transfer and transport of hordenine trough the intestinal barrier and blood-brain barrier was investigated using *in vitro* models. The Caco-2 monolayer model was applied to predict the absorption of orally administered hordenine, while the PBCEC monolayer system was used to analyze the BBB permeation. Both barrier systems were based on similar experimental setups as described under Materials and methods.

### Cytotoxicity screening of hordenine

In the first step the cytotoxicity of hordenine was screened to determine the maximum non-toxic concentration that will not impair the integrity of the cell monolayers. The application of hordenine at a concentration range from 0.1 μM to 100 μM did not reduce the viability of either Caco-2 cell or PBCEC compared to the respective negative controls (S6 Fig in S1 File). As a result, a hordenine concentration of 1 μM was chosen for the passive transport studies.

### Identification of hordenine sulfate in Caco-2 cells

On the one hand, initial transfer studies in the Caco-2 model showed that the amount of hordenine in the apical and basolateral compartments summed up was not 100%, indicating biotransformation of hordenine. On the other hand, the well-known metabolic activity of Caco-2 cells and the biotransformation of compounds observed in previous studies [18] motivated the investigation of the metabolism of hordenine.

Untargeted and targeted HPLC-HRMS analysis in positive and negative ionization mode was performed, revealing the phase-II metabolite hordenine sulfate in Caco-2 cells after 48 h incubation of hordenine. For the detected peak, *m*/*z* 244.0657, only negligible differences between the calculated and accurate masses (mass error 3.1 ppm) were determined, leading to the molecular formula [C_10_H_15_NO_4_S-H]^-^ for the deprotonated molecule (S7A Fig in S1 File). The isotopic pattern was used to tentatively identify hordenine sulfate. In addition to the ion [M-H]^-^ with *m*/*z* 244.0657 and the ^13^C isotope signal of hordenine sulfate ([M-H]^-^ + 1.0031 u) with *m*/*z* 245.0688, a signal with *m*/*z* 246.0618 was observed (S7B Fig in S1 File). The mass differences of 1.9961 u and a relative intensity of 4.5% compared to [M-H]^-^ are characteristic for the ^34^S isotope. The isotopic abundance of ^34^S is in the range of 3.96–4.77% in natural materials [24]. MS/HRMS experiments also indicate the sulfate conjugate of hordenine. The neutral loss of SO_3_ and the resulting fragment (*m*/*z* 164.1086) corresponding to hordenine were detected in negative ionization mode (S7C Fig in S1 File).

### Synthesis and structural elucidation of hordenine sulfate

To confirm the structure of the identified hordenine sulfate and to quantify the biotransformation rate of hordenine in the barrier-forming cells, hordenine sulfate was synthesized for the first time as described in Materials and Methods. After purification of the synthetic product by semipreparative HPLC in HILIC mode and UV detection, hordenine sulfate was obtained as a white powder with a yield of approximately 90% and a purity of 99% (HPLC-ELSD). The obtained substance was characterized by HRMS and MS/HRMS analysis as well as NMR spectroscopy and confirmed the proposed structure of hordenine sulfate.

The HRMS analysis of the synthesized compound revealed only a low mass error (2.0 ppm) between calculated and detected *m*/*z* for the deprotonated molecule with the formula [C_10_H_15_NO_4_S-H]^-^. The isotopic pattern with the characteristic ^34^S isotope signal and a relative intensity of 4.5% in comparison to [M-H]^-^ confirmed the sulfate moiety in the molecule (S8A Fig in S1 File). Moreover, MS/HRMS analysis of the synthesized substance with *m*/*z* 244.0654 revealed the neutral loss of SO_3_ to the resulting fragment *m*/*z* 164.1080 corresponding to hordenine (S8B Fig in S1 File). Thus, HRMS analysis and HPLC retention time of 2.8 min (S9 Fig in S1 File) of the synthesized hordenine sulfate were consistent with the data from hordenine sulfate in Caco-2 cells and allowed a clear identification.

The ^1^H- and ^13^C-NMR analysis of the synthesis product revealed clear signals for the sulfation of hordenine, which agreed well with the software-based prediction. Comparing the ^1^H-NMR spectra of the sulfated and unmodified hordenine, the major differences in the chemical shifts were found at the protons H-2 and H-6 (Δδ+0.4 ppm), indicating a representative downfield shift due to the *ortho* effect of the adjacent sulfate moiety (S10 Fig, S2 Table in S1 File). Further confirmation of the presence of the sulfate group was provided by the carbon shifts observed in the ^13^C NMR spectrum. A large upfield shift of Δδ-3.6 ppm was detected at the carbon bearing the sulfate moiety (C-9), and a downfield shift of the adjacent carbons was observed with respect to hordenine. The signals of the carbons C-2 und C-6 in *ortho* position and carbon C-4 in *para* position were shifted to a higher magnetic field with Δδ+5.50 ppm and Δδ+4.04 ppm, respectively (S11 Fig, S2 Table in S1 File). The characteristic up- und downfield displacements induced by a sulfate moiety agree with literature data [22, 25]. In addition to structure elucidation via NMR, the concentration of a hordenine sulfate reference solution later used for quantitation was evaluated using qNMR.

### Development of a quantitation method for hordenine and hordenine sulfate

**Fig 2:**
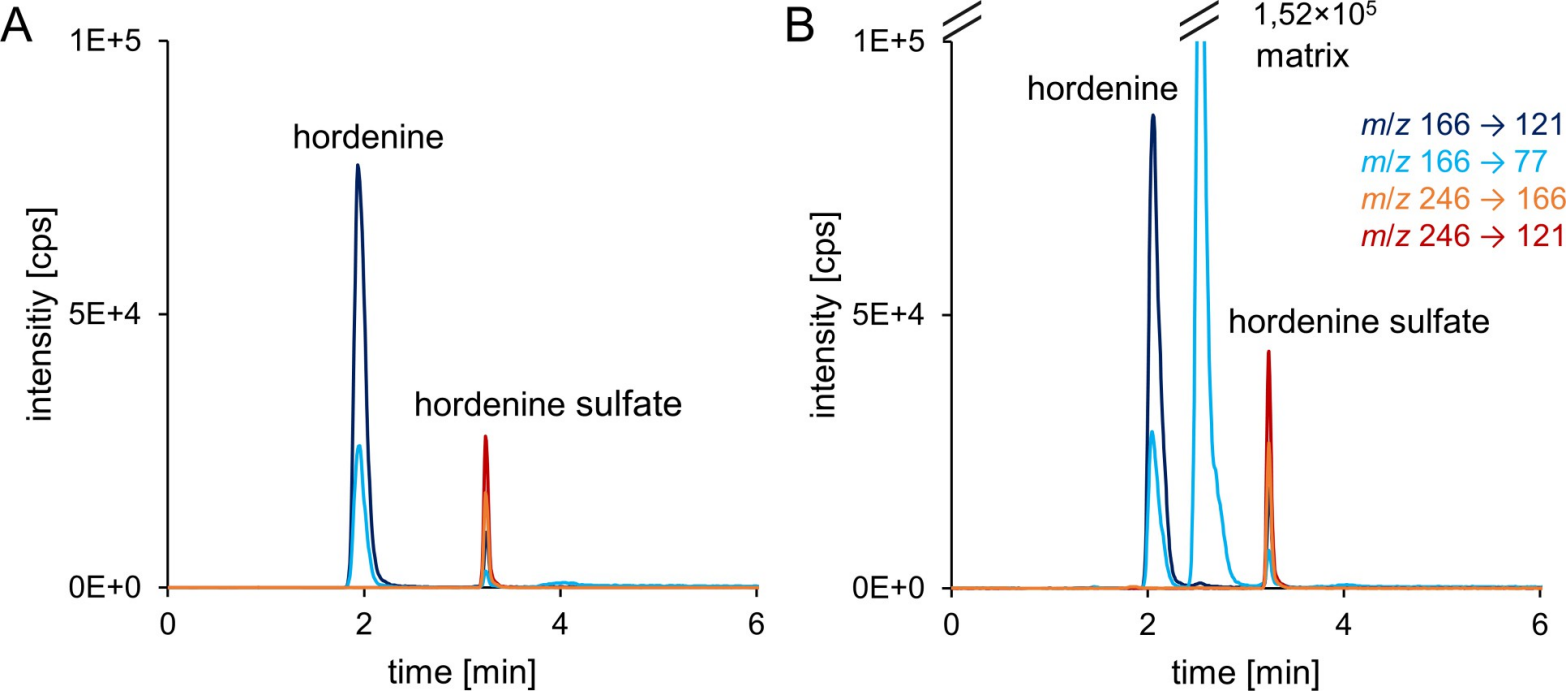
Extracted ion chromatograms of standard solution containing 500 nM hordenine and hordenine sulfate diluted in MeOH/H_2_O (1+19, *v*/*v*) (A) and in cell culture matrix (B). Both qualifier and quantifier MRM transitions are shown. The matrix signal (intensity: 1,52×10^5^ cps) is resulting from cell culture medium.

For simultaneous quantification of hordenine and hordenine sulfate in cell culture samples, a sensitive HPLC-MS/MS method was developed. Different column materials (C18, phenylhexyl, pentafluorophenyl, carbon) were investigated, but best chromatographic performance was obtained with a biphenylpropyl-based stationary phase (Fig 2A). The total run time was 10 min, with analytes eluting in the first 4 min and column re-equilibration in the last 5 min, allowing for high-throughput analysis.

The concentration of hordenine and hordenine sulfate was directly determined in cell culture medium without further extraction procedures. Therefore, matrix matched external calibration was used to compensate for matrix effects. This HPLC-MS/MS method enabled a rapid analysis to detect hordenine and its sulfate in cell culture samples in the low nanomolar range with an acceptable signal-to-noise ratio (S3 Table in S1 File).

### Barrier integrity of Caco-2 and PBCEC monolayer

**Fig 3:**
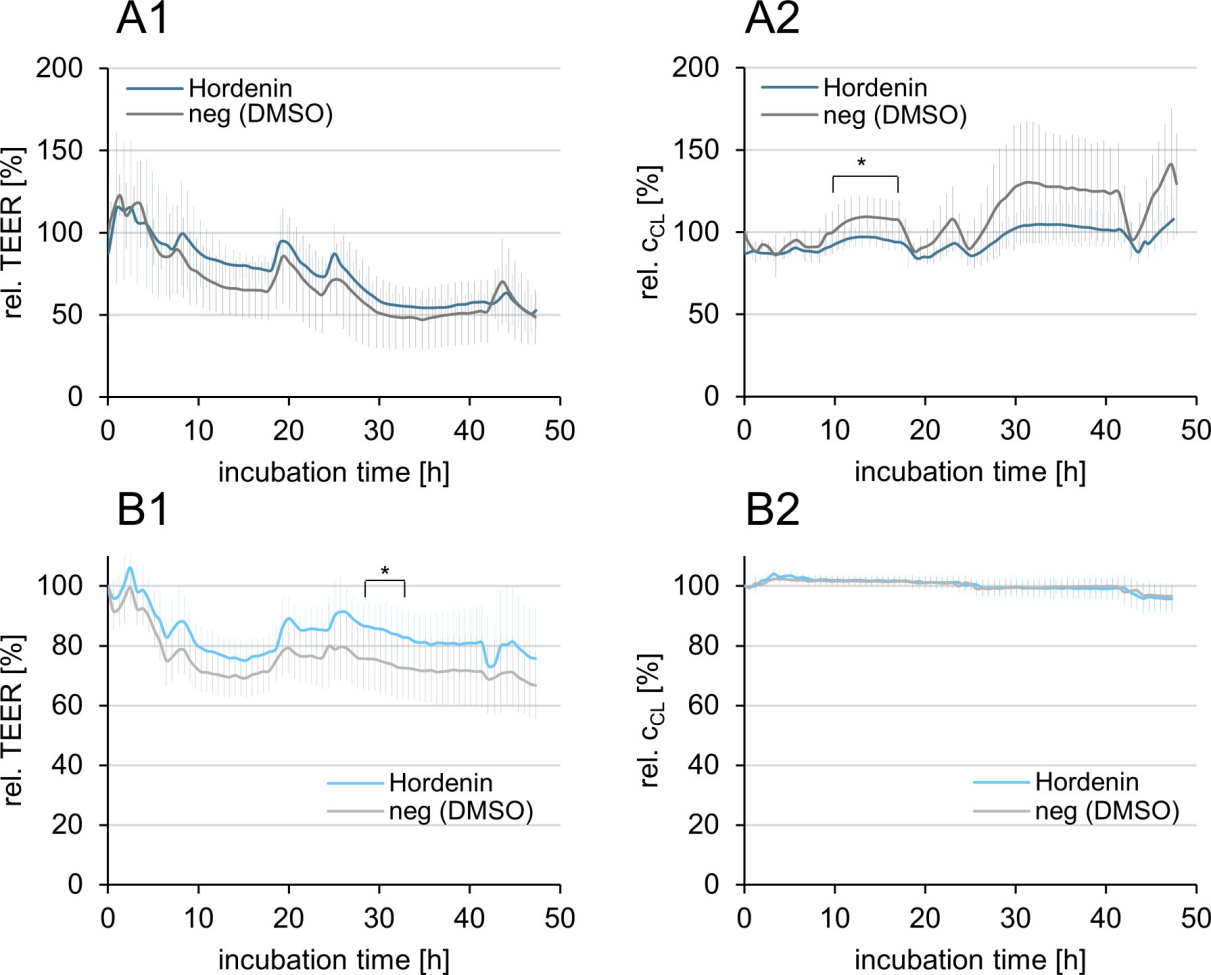
Relative transepithelial/transendothelial electrical resistance (TEER) and electrical capacitance (*c*_CL,_) of Caco-2 cells (A1/A2) and primary porcine brain capillary endothelial cells (PBCEC, B1/B2) incubated with 1 μM hordenine or solvent (neg, 0.1% DMSO) for 48 h (*n* = 3 × 3). The data are normalized to the initial values at the beginning of the transport studies and represented as means ± standard deviations. Significant differences (*p* < 0.05) between the test substances and the respective negative control were calculated in the Student’s *t*-test and marked with asterisks.

To analyze the barrier integrity of the cellular monolayers, transepithelial/transendothelial electrical resistance (TEER) and electrical capacitance (*c*_CL_) were monitored during transport studies. For the passive transfer experiment, the barrier-forming cells were cultured on permeable Transwell^®^ membranes and incubated with 1 μM hordenine in the apical compartment. Changes in barrier integrity were monitored over 48 h and are shown in Fig 3 as a percentage of the initial TEER and capacitance values.

In both the Caco-2 (Fig 3A1) and PBCEC BBB (Fig 3B1) models, hordenine did not impair the barrier integrity. The relative TEER values of cells incubated with hordenine were of the same order of magnitude as those of the respective negative controls, indicating that hordenine had no barrier influencing properties. At the end of the transport studies, approximately 50% for Caco-2 cells and 80% for PBCEC of the initial TEER values were maintained after 48 h treatment with 1 μM hordenine. Due to the long differentiation period of 21 d, Caco-2 cells were sensitive to incubation of compounds and sampling, resulting in an increase or decrease in TEER and thus high standard deviations. However, the high absolute TEER values of 1750 ± 260 Ω∙cm^2^ (solvent treated Caco-2 cells) and 1735 ± 530 Ω∙cm^2^ (hordenine treated Caco-2 cells) immediately before substance incubation indicated an intact Caco-2 monolayer suitable for transport studies [14, 18, 26] (S12A Fig in S1 File). The PBCEC monolayer showed absolute TEER values above 600 Ω∙cm^2^ at the beginning of the experiment, which is characteristic of an stable PBCEC barrier (S12B Fig in S1 File) [27]. Moreover, hordenine did not impair the confluency of the monolayer of the barrier-forming epithelial (Fig 3A2) and endothelial (Fig 3B2) cells as reflected in the constant *c*_CL_.

The permeability of Lucifer Yellow (LY) verified the integrity of the cell monolayers. After completion of the transport studies, LY was applied to cells previously treated with hordenine or with solvent (0.1% DMSO). The permeability coefficients *p*_c_ of LY were calculated according to equation 1 and equation 2 (S1 Equation in S1 File) and were in the same order of magnitude for cells incubated with solvent and for hordenine treated cells (Table 1). For the Caco-2 model, the LY values are comparable with the literature data of LY with *p*_*c*_ (LY) = 0.70 ± 0.10×10^−6^ cm/s [28] and the paracellular marker mannitol with *p*_*c*_ (mannitol) = 0.12 ± 0.05×10^−6^ cm/s [26] indicating tight Caco-2 monolayers with restricted paracellular diffusion.

**Table 1:** Permeability coefficients of Lucifer Yellow (LY) after 48 h preincubation with hordenine (1 μM) and solvent (0.1% DMSO) (*n* = 3 × 2).

Cell model	Preincubation with test compound	Permeability coefficient of LY *p*_c_ [10^−6^ cm/s]
Caco-2	Hordenine	0.40 ± 0.39
	Solvent (DMSO)	0.13 ± 0.09
BBB	Hordenine	0.79 ± 0.77
	Solvent (DMSO)	0.46 ± 0.35

The tightness of the PBCEC monolayer is often characterized by the permeability of the paracellular marker ^14^C-sucrose with *p*_*c*_ (^14^C-sucrose) = 1.0 ± 0.4 ×10^−6^ cm/s [29], which is comparable to LY (Table 1), indicating a intact endothelial barrier in the present study.

### Transfer and transport studies in the Caco-2 model

The Caco-2 monolayer model is a well-established tool for studies of intestinal transport mechanism and prediction of oral drug absorption [14, 15]. In this study, the passive transfer of hordenine from the intestinal “lumen” side (apical) to the “blood” side (basolateral) was investigated.

**Fig 4:**
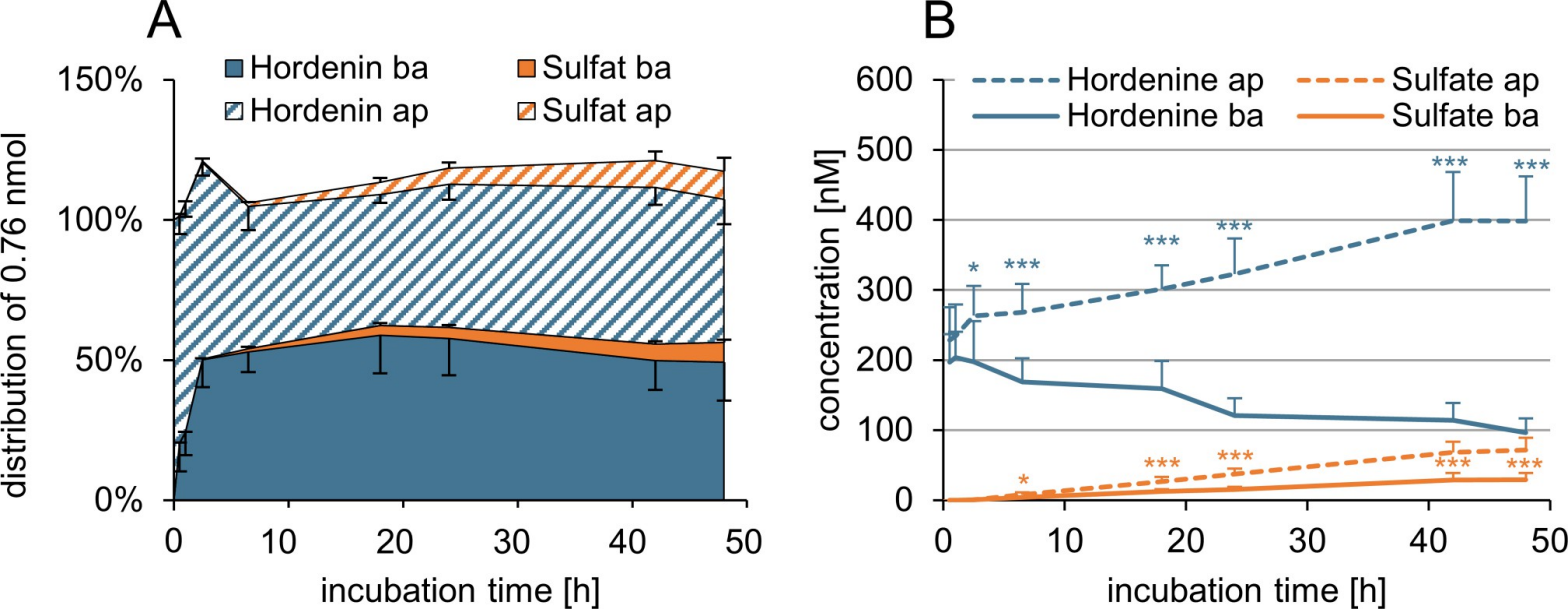
Passive transfer (A) and active transport (B) of hordenine through the Caco-2 monolayer over the course of 48 h. The passive transfer of 1 μM hordenine from apical (ap) to basolateral (ba) compartment was determined (*n* = 3 × 3). Hordenine and hordenine sulfate (abbreviated as sulfate) were quantified using HPLC-MS/MS and normalized to the initial amount (0.76 nmol) on the apical side. For active transport experiments equimolar concentrations of 200 nM hordenine were applied in the apical and basolateral compartment (*n* = 3 × 3). Data are presented as means ± standard deviations. Statistically significant differences between concentration in the two compartments are marked by asterisks (*: *p* ≤ 0.05; **: *p* ≤ 0.01; ***: *p* ≤ 0.001).

After cultivation and differentiation of Caco-2 cells, 1 μM hordenine was applied in the apical compartment of Transwell^®^ filter inserts for 48 h. Samples were withdrawn from the apical and basolateral compartments at eight points in time and hordenine as well as hordenine sulfate were quantified by HPLC-MS/MS. Fig 4A shows the time-dependent distribution of hordenine and its metabolite hordenine sulfate in both compartments. The transfer of hordenine across the intestinal barrier was very fast. Already after 1 h, 24 ± 8% (0.5 h: 20 ± 10%) of the applied hordenine was detected in the basolateral compartment, increased to 50 ± 10% after 2.5 h and reached a plateau up to the end of the transport study (48 h: 49 ± 14%).

After 6.5 h, first traces of hordenine sulfate were detected in both compartments, increasing to 10 ± 5% in the apical and 7 ± 1% in the basolateral compartments after 48 h (Fig 4A). The total amount of hordenine recovered in the system, i.e., the sum of hordenine and hordenine sulfate in both compartments, was close to 100% throughout the transport study. As hordenine was transferred through the intestinal epithelium after only a few minutes and hordenine sulfate was first detected after 6.5 h, a permeability coefficient of *p*_c_ = 99.8 ± 18.2 ×10^−6^ cm/s was calculated for hordenine with linear regression between 30 and 60 min, when no hordenine was metabolized.

In addition to passive transfer across the intestinal barrier, active transport was analyzed by applying hordenine in equimolar concentrations of 200 nM in both compartments of the Transwell^®^ filter system. In the case of active transport mechanism, the test compound would accumulate on one side. Fig 4B shows the results of the active transport study of hordenine in the Caco-2 model. Hordenine concentrations increased continuously in the apical compartment and decreased simultaneously in the basolateral compartment as the incubation period progressed. The difference between both compartments for hordenine was statistically significant after 2.5 h (*p* < 0.05), 6.5–48 h (*p* < 0.001). At the end of the experiment, an enrichment of hordenine with 399 ± 64 nM was detected in the apical compartment, whereas 100 ± 20 nM hordenine were quantified in the basolateral compartment, indicating an efflux of hordenine from the “blood”-side back to the “lumen”-side. Moreover, hordenine was metabolized to hordenine sulfate and was detected mainly in the apical compartment with 71 ± 18 nM, whereas the concentration of hordenine sulfate in the basolateral compartment was 30 ± 9 nM after 48 h. The enrichment of hordenine sulfate by approximately a factor two in the apical compartment may lead to the assumption that the sulfate was actively transported to the apical side of the membrane. However, if the dilution factor of 2.17 resulting from the different volumes of the two compartments is considered, the molar amount of hordenine sulfate on both sides was very similar.

### Transfer and transport studies in the BBB model

**Fig 5:**
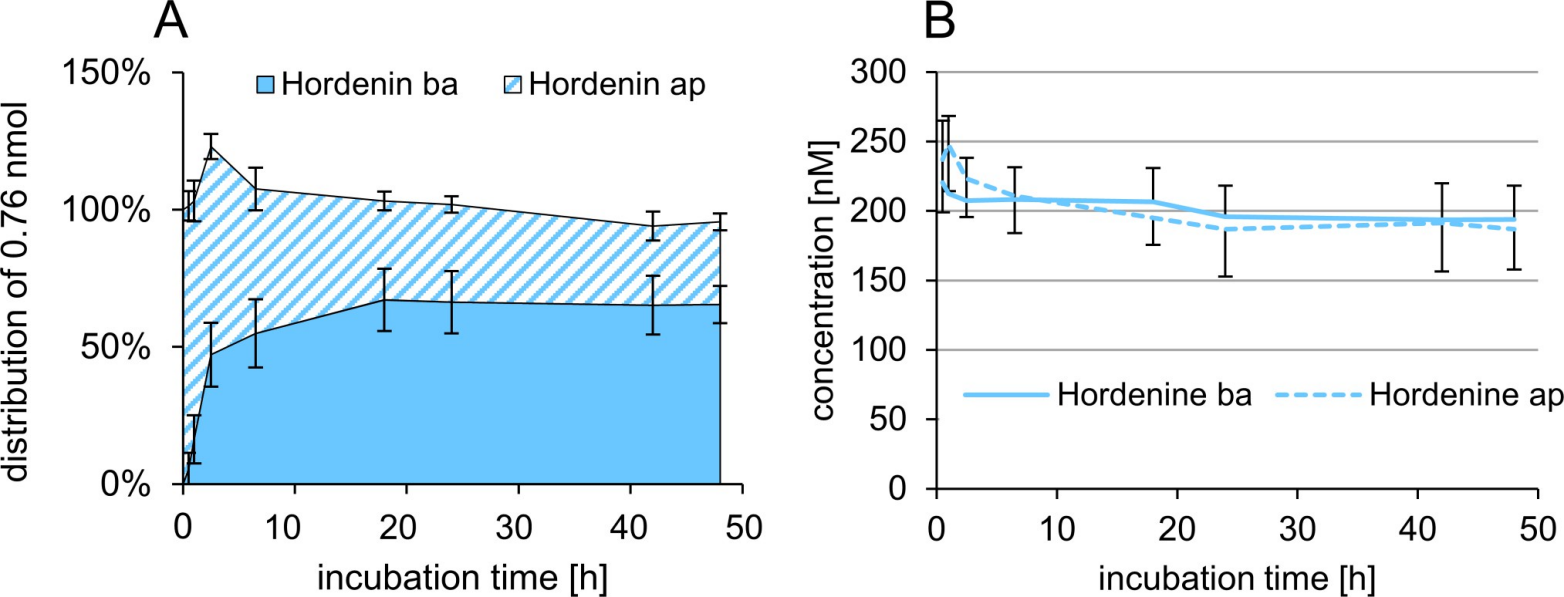
Passive transfer (A) and active transport (B) of hordenine through the PBCEC monolayer. The passive transfer of hordenine from apical (ap) to basolateral (ba) compartment was determined after apical application of 1 μM hordenine (*n* = 3 × 3). Hordenine was quantified using HPLC-MS/MS and normalized to the initial amount (0.76 nmol) on the apical side. The active transport experiments based on the recovery of 200 nM hordenine in the apical and basolateral compartment after incubation of equimolar concentrations in both compartments (*n* = 3 × 3). Data are presented as means ± standard deviations.

The passive transfer of hordenine through the PBCEC monolayer was analyzed using the same sampling and quantitation pattern as in the Caco-2 model. The resulting time-dependent distribution of hordenine in both compartments of the filter system after apical application of 1 μM is illustrated in Fig 5A. The fast transfer of hordenine across the BBB is evident by the fact that 16 ± 9% of the incubated hordenine was detected in the basolateral compartment after only 1 h. Permeation of hordenine increased to 47 ± 12% after 2.5 h and reached saturation at approximately 65% from 18 h to 48 h. A permeability coefficient of *p*_c_ = 55.4 ± 13.1 ×10^−6^ cm/s (30–60 min) was calculated for the BBB model. The metabolism of hordenine in PBCEC was also analyzed by HPLC-HRMS, and no metabolic conversion to hordenine sulfate or further biotransformation process was observed.

The results of the active transport studies in the BBB model are shown in Fig 5B. Hordenine was applied in equimolar concentrations of 200 nM on both sides of the PBCEC monolayer. After 48 h, 187 ± 29 nM hordenine was detected in the apical and 198 ± 24 nM hordenine in the basolateral compartment. The absence of an enrichment in any compartment suggested that an active transport of hordenine through the PBCEC monolayer is unlikely.

## Discussion

High oral bioavailability and rapid permeation of the BBB are prerequisites for CNS-active compounds to reach their side of action after oral uptake. In this context, investigations on the transfer and transport properties of the bioactive and potentially dopaminergic hordenine through the intestinal barrier and BBB are of particular interest.

Hordenine had neither cytotoxic nor barrier-impairing effects on Caco-2 and PBCEC monolayers in concentrations up to 100 μM, thus the focus of this study was on the transport characteristics. Comparing both cell culture systems, similar transfer kinetics of hordenine were observed. The transfer of hordenine across the Caco-2 monolayer was slightly faster than through the PBCEC monolayer, as reflected by a higher permeability coefficient (Table 2). The main difference was the efflux transport of hordenine, which was detected only in the Caco-2 model. A good correlation between permeability studies of seven test substances using the Caco-2 model and the PBCEC monolayers was also found in a previous study. Differences in permeability coefficients were only observed for substances with special transport properties [27].

**Table 2:** Permeability coefficients of hordenine calculated in the Caco-2 and BBB model in comparison to test compounds with their literature reference. Permeation of hordenine from apical to basolateral compartment of the Caco-2 or PBCEC monolayer with apical application of 1 μM hordenine (passive transfer study, *n* = 3×3).

Cell model	Test compound	Reference	Permeability coefficient *p*_c_ [10^−6^ cm/s]
Caco-2	Hordenine	present study	99.8 ± 18.2
	Antipyrine	[30]	76.7 ± 3.6
	Ibuprofen	[30]	60.1 ± 3.5
	Ephedrine	[31]	10.2 ± 0.6
BBB	Hordenine	present study	55.4 ± 13.1
	Caffeine	[29]	62.4 ± 7.7
	Diazepam	[27]	53.0± 3.0

### Intestinal absorption in Caco-2 model

The results of the passive transfer studies using the Caco-2 model indicated a very fast transfer of hordenine across the intestinal barrier. After only 30 min, 20 ± 10% of the applied hordenine was detected in the basolateral compartment. Due to the moderate hydrophilicity of hordenine, various transport mechanisms are conceivable. Transport of small hydrophilic drugs via the paracellular pathway is restricted by tight junctions formed between neighboring epithelial cells [15, 32]. As TEER increases based on the integrity of tight junctions, the high absolute TEER values of the Caco-2 monolayers indicated a negligible contribution of paracellular diffusion to the overall rapid transfer across the monolayer. Moreover, the permeability coefficient of hordenine with *p*_c_ = 99.8 ± 18.2 ×10^−6^ cm/s was much higher than that of the paracellular marker LY (*p*_c_ = 0.40 ± 0.39 ×10^−6^ cm/s), also suggesting that hordenine was not transferred via the paracellular pathway. Most drugs that are rapidly absorbed after oral administration are transferred by the passive transcellular route and have high permeability coefficients (*p* > 1 × 10^−6^ cm/s) [14, 15]. If the high *p*_c_ of hordenine in the Caco-2 model is transferred to *in vivo* conditions, high intestinal permeability and bioavailability can be expected. A good correlation between experimental permeability values in the Caco-2 cell assay with the extent of human absorption was shown in several studies. For example, the drugs antipyrine and ibuprofen with *p*_c_ of the same order of magnitude (Table 2) were completely intestinal absorbed *in vivo* [30]. For ephedrine, a phenethylamine with similar structure as hordenine, a much lower *p*_c_ (Table 2) was determined, but an absorption rate of 90% was still observed *in vivo*. This basic drug was transferred mainly by the transcellular route, although the proportion of unionized species at pH 7.2 and pH 5.4 was very low [31].

In addition to passive diffusion, drugs can be also transported through the intestinal epithelium by carrier-mediated transport [33]. Initial proposals are based on the structural similarity of hordenine to dopamine and may include active transporter that are mainly involved in the intestinal uptake of drugs. For example, absorption of levodopa, a precursor of dopamine, is mediated by several amino acid transporters located at the apical and basolateral enterocyte membranes [34]. In Caco-2 cells, a levodopa uptake system has been described that is also responsible for the efflux of newly formed dopamine from intracellular levodopa to the apical and basolateral sides [35]. Another transporter that could be considered for active transport of hordenine would be an organic cation transporter (OCT) assuming that hordenine is probably present as a cation under physiological conditions. For tyramine, the biosynthetic precursor of hordenine, an OCT-2 in the apical membrane and a Na^+^-dependent, atropine-sensitive active transporter in the basolateral membrane of Caco-2 cells were described. This transport system regulated the influx and efflux of tyramine across the intestinal epithelium [36].

**Fig 6:**
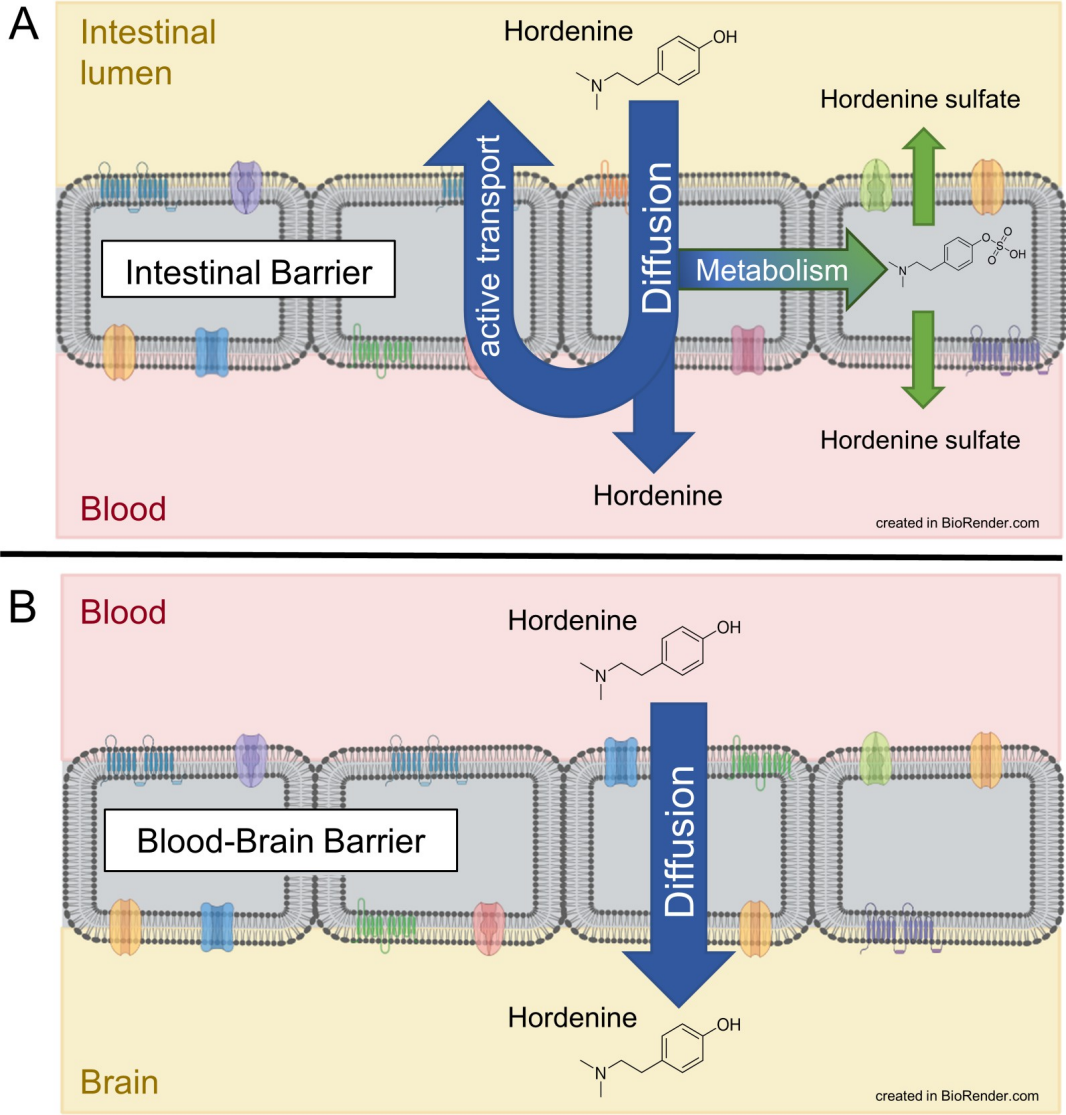
Schematic illustration of transport and metabolism of hordenine through the intestinal barrier (A) and the blood-brain barrier (B).

Results of our active transport studies using the Caco-2 model showed an enrichment of hordenine in the apical compartment, indicating an efflux of hordenine from the bloodstream back to the intestinal lumen (Fig 6A). In Caco-2 cells, drug efflux is mediated mainly by ATP binding cassette (ABC) efflux transporters such as P-glycoprotein (P-gp, ABCB1 gene), breast cancer resistance protein (BCRP, ABCG2 gene), and multidrug-resistance proteins (MRP), specifically MRP2, which are expressed at the apical membrane [37, 38]. Whether hordenine is a substrate of these efflux transporters in Caco-2 cells is unknown. However, the results of the active transport studies in the BBB model gave a hint, as no involvement of transport proteins in the transport of hordenine through the PBCEC monolayer has been shown (Fig 6B).

In principle, the efflux transporter P-gp, BCRP and several MRPs are also expressed in the BBB, with P-gp and BCRP localized in the luminal membrane [16]. Even in PBCEC, the expression of P-gp and BCRP has been detected [39]. If these transporter with a broad substrate specificity play a role in the transport of hordenine across the cellular barriers, they would likely have also contributed to efflux of hordenine in the PBCEC model and not only in the Caco-2 model. Therefore, comparison of the transporter expression of both cell monolayers and the results of active transport studies suggest that the influence of P-pg or BCRP on hordenine efflux seems less likely than MRPs.

The involvement of efflux transport proteins seems to actively reduce the high transfer rates of hordenine across the intestinal barrier, resulting in a reduction of bioavailability. At the end of the transfer studies, permeation of 49 ± 14% was determined in the Caco-2 model compared with a permeation of 65 ± 7% in the PBCEC monolayer. Thus, the rapid efflux of hordenine in the Caco-2 model resulted in a shift of the equilibrium concentration to the apical side compared to the BBB model. Consequently, the intestinal efflux of hordenine might cause the low bioavailability of hordenine observed *in vivo* and the associated low levels of hordenine in the blood after oral administration [12, 13]. Elucidation of the transfer mechanism of hordenine in further detail and characterization of specific transport proteins in the used Caco-2 cells might be interesting subjects for further studies.

In the used Caco-2 model, the metabolite hordenine sulfate was detected in the passive and active transport studies, suggesting that bioavailability may also be reduced by biotransformation of hordenine. First traces of hordenine sulfate were analyzed after 6.5 h of incubation of hordenine in Caco-2 cells, which were enriched to 10 ± 5% after 48 h. Rapid absorption rates [2, 40] and biotransformation of hordenine have also been observed *in vivo* [12, 13]. After consumption of beer containing hordenine, the maximum plasma concentration of free hordenine was detected in four volunteers within 30 min. Thereafter, the hordenine concentration decreased rapidly, and hordenine conjugates were observed simultaneously, indicating biotransformation of hordenine immediately after absorption. Hordenine sulfate appeared to be the major metabolite of hordenine formed directly after oral exposure as the hordenine sulfate content exceeded that of hordenine glucuronide, which was identified as additional metabolite *in vivo* [12]. The fast sulfation of hordenine both *in vivo* and in Caco-2 cells indicates a first pass effect in the intestinal epithelium during intestinal absorption. However, other tissues might also be capable of hordenine sulfation. In contrast, glucuronidation of hordenine probably occurs in the liver, as hordenine glucuronide levels in human plasma increased only after a prolonged period of time and, moreover, hordenine glucuronide was not detected in Caco-2 cells. However, previous studies have shown that several factors, such as differentiation and passage number of Caco-2 cells, affects the expression levels of UDP-glucuronosyltransferases [41]. Therefore, glucuronidation activity may be different among different Caco-2 cell cultures, and glucuronidation of hordenine cannot be generally excluded.

Furthermore, the preliminary biokinetic studies after oral administration of hordenine in humans showed an elimination half-live of free hordenine of approximately t_½_ = 60 min [12, 13] and maximum urinary excretion rates after 2-3.5 h [12]. However, it is striking in this study that hordenine conjugates were still detectable in the urine after 24 h in one of four participants. In one other volunteer, only about 10% of the ingested hordenine was determined in the form of sulfate or glucoronate. Our results of the transfer studies using the Caco-2 model confirmed the metabolism to hordenine sulfate and indicated a rapid intestinal absorption, potentially leading to rapid excretion of hordenine and its conjugates. However, the efflux of hordenine from bloodstream back to the intestinal lumen observed in the present study could decrease urinary elimination rates. On the other hand, distribution of hordenine in highly perfused organs and tissues, including the brain, is conceivable, as indicated by the preliminary pharmacokinetic study of Sobiech and co-workers [13]. Considering the agonistic activity of hordenine towards the D2R, distribution in the brain and especially the permeability through the BBB is of particular interest.

### Permeability through the BBB (PBCEC model)

The fast transfer of hordenine through the PBCEC monolayer indicates a rapid penetration of hordenine from the circulating blood stream into the brain. The permeation rate of hordenine with a permeability coefficient of *p*_c_ = 55.4 ± 13.1 ×10^-6^ cm/s can be classified as high. Previous studies showed a similar permeability through the BBB for caffeine (Table 2) using the PBCEC model and a corresponding brain uptake index of 103% *in vivo* [29]. The psychoactive drug diazepam was predicted to have a high BBB permeation with a *p*_c_ value, which is in a similar range to hordenine and caffeine (Table 2) [27]. A previous study of Könczöl and co-workers using the parallel artificial membrane permeability assay (PAMPA)-BBB predicted a good BBB-permeability potential for hordenine. In general, the cell-free permeation test is a useful tool for screening compounds for brain penetration in CNS drug discovery. However, this approach involves only passive diffusion processes across the BBB and not transporter-mediated absorption, such as active uptake or efflux. Furthermore, PAMPA neglects effects of metabolism on the BBB permeation [42, 43]. Therefore, it was of particular interest to investigate the permeability of hordenine through a cell membrane and further elucidate the transfer mechanism through the BBB *in vitro*.

Results of the active transport studies using the PBCEC model showed no efflux of hordenine from the brain back into the circulation and *vice versa*, suggesting that transport proteins play a minor role in the brain penetration of hordenine. In the absence of efflux processes, the transfer rate in the BBB model should theoretically be higher than in the Caco-2 model. However, contrary to expectations, a lower permeability coefficient *p*_*c*_ was detected. The larger surface area of the intestinal epithelium formed by a brush border at the apical membrane of Caco-2 cells, might be a possible reason for the faster transfer of hordenine in contrast to the unfold PBCEC membrane [27].

Moreover, biotransformation of hordenine in PBCEC was not detected, resulting in no reduction of the high exposure of hordenine into the brain. The agonistic activity of hordenine towards the D2R has already been described [6]. However, penetration of hordenine through the BBB is crucial for the activation of dopaminergic pathways in the brain. Two *in vivo* consumption studies showed that the plasma levels of hordenine after oral administration were probably too low to directly interact with D2R [12, 13]. However, a stronger accumulation of hordenine in the brain increases the probability of hordenine interactions with D2R.

If the first pass effect of hordenine after oral administration is to be circumvented to use hordenine as a pharmaceutical D2R activator, an alternative route of administration, e.g., intravenous or nasal, may be considered as previously described for dopamine. Since dopamine has poor oral bioavailability and is unable to pass the BBB, transport of dopamine from the olfactory mucosa into brain allowed improved targeted delivery of dopamine into the CNS. In several animal species, including mice, rats, rhesus monkeys, and beagle dogs, increased uptake of dopamine into the CNS after nasal administration has already been described [44–47]. Intranasal administration may also be an attractive noninvasive route for hordenine delivery because of its many advantages, including avoidance of first pass-metabolism. A second optional administration route may be the inhalation to bypass the gastrointestinal tract, as described for levodopa administration [48].

## Conclusion

In conclusion, our data characterize the transfer and transport properties of bioactive hordenine across biological barriers and to extend the knowledge of its pharmacokinetics. Hordenine was transferred very quickly through the Caco-2 monolayer, indicating rapid absorption in the small intestine after oral administration. However, the observed efflux transport of hordenine from the bloodstream back into the intestinal lumen and the metabolism to hordenine sulfate in intestinal epithelial cells may reduce the bioavailability. The synthesis of hordenine sulfate served to assess the biotransformation rate of hordenine in the Caco-2 model and provided an approach for the preparation of its analytical standard for future use in physiological samples. In addition, hordenine was able to rapidly penetrate the BBB *in vitro*, comparable to CNS active drugs. The absence of efflux transport and the possibility of hordenine accumulation in the brain increases the probability that sufficient levels of hordenine could lead to an interaction with D2R. Thus, a pharmaceutical approach with effective dosing via the nasal or intravenous route of administration might lead to higher brain uptake of hordenine and stronger D2R interaction. In this context, it would be interesting to know whether hordenine as a D2R agonist has therapeutic potential for the treatment of CNS disorders.

## Supporting information

S1 Dataset HRMS data of synthesized hordenine sulfate recorded with an qTOF mass spectrometer in negative ionization mode. (XLSX)

S1 File (DOCX)
