# Supplementary material for: Permeability of dopamine D2 receptor agonist hordenine across the intestinal and blood-brain barrier in vitro
Source: PLoS One. 2022 Jun 16;17(6):e0269486. doi: 10.1371/journal.pone.0269486 (PMC9202863; doi:10.1371/journal.pone.0269486)
Supplement: S1 File — (DOCX) [file pone.0269486.s002.docx]

**Supporting Information**

**Permeability of dopamine D2 receptor agonist hordenine across the intestinal and blood-brain barrier *in vitro***

Maria Hahn^1^, Viktoria Lindemann ^1^, Matthias Behrens^1^, Dennis Mulac^2^, Klaus Langer^2^, Melanie Esselen^1^, Hans-Ulrich Humpf^1*^

^1^Institute of Food Chemistry, Westfälische Wilhelms-Universität Münster, Corrensstr. 45, 48149 Münster, Germany

^2^Institute of Pharmaceutical Technology and Biopharmacy, Westfälische Wilhelms-Universität Münster, Corrensstr. 48, 48149 Münster, Germany

*[humpf@wwu.de](mailto:humpf@wwu.de)

**Permeability coefficients**

$$\text{ }\text{p}\text{ }\left[ \frac{\text{cm}}{\text{s}} \right]\text{=}\text{ }\frac{{\text{d}\text{c}}_{\text{bas}}\left[ \text{\%} \right]}{\text{d}\text{t}\text{ }\left[ \text{s} \right]}\text{ × }\frac{\text{V}_{\text{ap}}\text{[}\text{cm}^{\text{3}}\text{]}}{\text{A}\text{ }\text{[}\text{cm}^{\text{2}}\text{]}\text{ × }\text{c}_{\text{0 h,ap}}\text{[\%]}}$$

(1)

$\text{ p }\text{[}\text{cm}\text{/}\text{s}\text{]}$: permeability coefficient

$\frac{{\text{d}\text{c}}_{\text{bas}}\text{[\%]}}{\text{d}\text{t}\text{ [s]}}$: slope of amount of test compound in basolateral compartment versus time curve

$\text{V}_{\text{ap}}\text{[}\text{cm}^{\text{3}}$]: volume of apical compartment

$\text{A}\text{ }\text{[}\text{cm}^{\text{2}}\text{]}$: surface area of filter inserts

$\text{c}_{\text{0 h,ap}}\text{[\%]}$: initial concentration in apical compartment at beginning of experiment

$$\frac{\text{1}}{\text{p}_{\text{c}}\left[ \frac{\text{cm}}{\text{s}} \right]}\text{ = }\frac{\text{1}}{\text{p}_{\text{c+m}}\left[ \frac{\text{cm}}{\text{s}} \right]} \text{- }\frac{\text{1}}{\text{p}_{\text{m}}\left[ \frac{\text{cm}}{\text{s}} \right]}$$

(2)

$\text{p}_{\text{c}}\left[ \frac{\text{cm}}{\text{s}} \right]$: corrected permeability coefficient of test compound

$\text{p}_{\text{c+m}}\left[ \frac{\text{cm}}{\text{s}} \right]$: permeability coefficient of test compound in transport experiment

$\text{p}_{\text{m}}\left[ \frac{\text{cm}}{\text{s}} \right]$: permeability coefficient of cell-free filter membrane

**S1 Equation. Equation (1) and (2) used for calculation of the permeability coefficients.**

**S1 Table. Instrument and method parameters for the purity measurement of hordenine sulfate by HPLC-UV-ELSD.**

| **LC-UV system** | Jasco (Jasco, Groß-Umstadt, Germany) | | |
| --- | --- | --- | --- |
| **Column** | TSKgel^®^ amide-80 column (7.8 mm id x 300 mm, particle size 10 µm, Tosoh Bioscience, Griesheim, Germany) | | |
| **Flow rate** | 2 mL/min | | |
| **Mobile phase** | ACN + 0.01% FA (**A**), H_2_O + 0.01%FA (**B**) | | |
| **Gradient** | time [min] | **A**[%] | **B**[%] |
|  | 0.0 | 65 | 35 |
|  | 0.5 | 65 | 35 |
|  | 13.0 | 5 | 95 |
|  | 14.0 | 5 | 95 |
|  | 15.0 | 65 | 35 |
|  | 30.0 | 65 | 35 |
| **UV Detection wavelength** | 266 nm | | |
| **ELSD** | Shimadzu (Duisburg, Germany), temperature 50 °C, pressurized air 350 KPa | | |

ACN, acetonitrile; FA, formic acid; id: inner diameter

**HRMS data of hordenine sulfate**

HRMS: *m/z* 244.0654 [M-H]^-^, calculated for [C_10_H_15_NO_4_S-H]^-^: *m/z* 244.0649. MS/HRMS (CID, Auto MS/MS): *m/z* (%): 164.1080 (32), 119.0503 (3), 106.0427 (100), 79.9579 (41).

**S1 Dataset. HRMS data of synthesized hordenine sulfate recorded with an qTOF mass spectrometer in negative ionization mode.**

**NMR spectra of hordenine sulfate**

^1^H NMR (600 MHz, DMSO-*d*_6_, referenced to solvent signal δ_H_ = 2.5 ppm): δ (ppm) = 2.25 (6H, s, H‑9+H‑10; 2×CH_3_), 2.53 (2H, m, H‑8; CH_2_), 2.67 (2H, m, *J*=7.1 Hz;8.4 Hz, H-7; CH_2_), 7.05 (2H, d, *J*=8.5 Hz, H‑2+H‑6; 2×CH), 7.10 (2H, d, *J*=8.5 Hz, H‑3+H‑5; CH).

**S1 Fig. ^1^H NMR spectrum of hordenine sulfate obtained in DMSO‑*d*_6_ at 600 MHz.**

^13^C NMR (150 MHz, DMSO-*d*_6_, referenced to solvent signal δ_C_ = 39.5 ppm): δ (ppm) = 32.17 (C‑7), 44.69 (C‑9+C‑10), 60.56 (C‑8), 120.46 (C‑2+C‑6), 128.79 (C‑3+C‑5), 134.47 (C‑4), 151.73 (C‑1).

**S2 Fig. ^13^C NMR spectrum of hordenine sulfate obtained in DMSO‑*d*_6_ at 150 MHz.**

**S3 Fig. HMBC NMR spectrum of hordenine sulfate obtained in DMSO‑*d*_6_.**

**S4 Fig. HSQC NMR spectrum of hordenine sulfate obtained in DMSO‑*d*_6_.**

**qNMR spectra of hordenine sulfate**

^1^H NMR (600 MHz, DMSO-*d*_6_, referenced to solvent signal δ_H_ = 2.5 ppm): δ (ppm) = 1.12 (6H, d, *J*=6.9 Hz, T‑H9+T‑H10; 2×CH_3_), 2.16 (3H, s, T‑H7; CH_3_), 2.19 (6H, s, S‑H9+S‑H10; 2×CH_3_), 2.45 (2H, dd, *J*=8.8 Hz, 6.7 Hz, S‑H8, CH_2_), 2.65 (2H, dd, *J*=8.9 Hz, 6.7 Hz, S‑H7, CH_2_), 3.19 (1H, hept, *J*=6.8 Hz, T‑H8; CH), 6.54 (1H, dd, *J*=7.7 Hz, 1.7 Hz, T‑H6, CH), 6.56 (1H, d, *J*=1.7 Hz, T‑H4, CH), 6.95 (1H, d, *J*=7.7 Hz, T‑H3, CH), 7.05 (2H, m, S‑H2+S‑H6, CH), 7.09 (2H, m, S‑H3+S‑H5, CH), 9.07 (1H, s, T‑H11, OH).

**S5 Fig.** **^1^H qNMR spectrum of hordenine sulfate obtained in DMSO‑*d*_6_ at 600 MHz.** Thymol was used as internal standard. ^1^H signals of hordenine sulfate are abbreviated as “S” and signals of thymol as “T”.

**Viability assay**

**S6 Fig. Relative viability of Caco-2 cells and PBCEC after incubation with hordenine for 48 h.** The resazurin reduction assay (*n* = 3 × 6) was performed for Caco‑2 cells and the CCK‑8 test (*n* = 3 × 6) was performed for PBCEC. DMSO (30%, Caco‑2) and T‑2 toxin (10 µM, PBCEC) served as positive controls (pos). Data are displayed as means ± standard deviations. Statistically significant differences compared to solvent-treated control (neg, 1% DMSO) calculated with Student’s *t*-test and marked with asterisks (*: *p*≤0.05; ***: *p*≤0.001).

**HPLC-HRMS analysis of hordenine sulfate**

**S7 Fig. HRMS spectrum at the retention time of hordenine sulfate in Caco‑2 cells (A) and isotopic pattern of hordenine sulfate in the same cell culture sample (B, zoomed view).** MS/HRMS fragmentation spectrum of the [M-H]^-^ adduct of hordenine sulfate (*m*/*z* 244.0657) in Caco‑2 cells (C).

**S8 Fig. HRMS spectrum of synthesized hordenine sulfate with isotopic pattern (A, zoomed view) and corresponding MS/HRMS fragmentation spectrum of the [M-H]^-^ adduct of hordenine sulfate (*m*/*z* 244.0654) (B).**

**S9 Fig. Combined extracted ion HPLC-qTOF-HRMS chromatograms of hordenine sulfate in Caco‑2 cells ([M-H]^-^ *m*/*z* 244.0657) and synthesized hordenine sulfate ([M-H]^-^ *m*/*z* 244.0654).** The Caco-2 cell sample was concentrated twofold, while 1 µM synthesized hordenine sulfate solution was used for HRMS analysis.

**NMR spectra of hordenine and hordenine sulfate in comparison**

**S10 Fig. ^1^H NMR spectrum of hordenine (A) and hordenine sulfate (B) obtained in DMSO‑*d*_6_ at 600 MHz.** ^1^H signals of hordenine are abbreviated as “H” and signals of hordenine sulfate as “S”. The downfield shift at S-H2 and S-H6 induced by the sulfate group is marked with a red arrow (*: signal of DMSO‑*d*_6_ at 3.3 ppm).

**S2 Table. ^1^H and ^13^C NMR data obtained for hordenine and hordenine sulfate determined in DMSO‑*d*_6_ and relative changes in chemical shifts, expressed as *δ*_Sulfate_ – *δ*_Hordenine_.**

|  | **Hordenine** | | **Hordenine sulfate** | | **^1^H shift** | **^13^C shift** |
| --- | --- | --- | --- | --- | --- | --- |
| position | *δ*(^1^H) [ppm] | *δ*(^13^C) [ppm] | *δ*(^1^H) [ppm] | *δ*(^13^C) [ppm] | *δ*(^1^H)_Sulfate_ – *δ*(^1^H)_Hord_ [ppm] | *δ*(^13^C)_Sulfate_ – *δ*(^13^C)_Hord_ [ppm] |
| 9+10 | 2.15 | 45.13 | 2.25 | 44.68 | +0.10 | -0.45 |
| 8 | 2.36 | 61.36 | 2.53 | 60.56 | +0.17 | -0.80 |
| 7 | 2.56 | 32.53 | 2.67 | 32.17 | +0.11 | -0.36 |
| 4 | - | 130.45 | - | 134.47 | - | **+4.02** |
| 3+5 | 6.98 | 129.38 | 7.10 | 128.79 | +0.12 | -0.59 |
| 2+6 | 6.64 | 114.96 | 7.05 | 120.46 | **+0.41** | **+5.50** |
| 1 | - | 155.36 | - | 151.73 | - | **-3.63** |

**S11 Fig. ^13^C NMR spectrum of hordenine (A) and hordenine sulfate (B) obtained in DMSO‑*d*_6_ at 150 MHz.** ^13^C signals of hordenine are abbreviated as “H” and signals of hordenine sulfate as “S”. The upfield displacement at S-C1 and the downfield shift at S‑C2 + S‑C6 and S‑C4 was produced by the sulfate group is marked with red arrows (*: unknown signal).

**Quantitation of hordenine and hordenine sulfate in cell culture samples**

**S3 Table**. **Calculated signal to noise ratio (S/N) for different concentrations of hordenine and hordenine sulfate diluted in HBSS buffer**.

| **Quantifier** | **Concentration [nM]** | **Calculated S/N** |
| --- | --- | --- |
| **Hordenine**  *m*/*z* 166 → 121 | 1 | 37 |
|  | 2 | 40.5 |
|  | 5 | 57.2 |
| **Hordenine sulfate**  *m*/*z* 246 → 121 | 1 | 13.5 |
|  | 2 | 22.5 |
|  | 5 | 42.5 |

**Barrier integrity of Caco‑2 and PBCEC monolayer**

**S12 Fig. Absolute transepithelial/transendothelial electrical resistance (TEER) of Caco-2 cells (A) and primary porcine brain capillary endothelial cells (PBCEC, B) incubated with 1 µM hordenine or solvent (neg, 0.1% DMSO) for 48 h (*n* = 3 × 3).** The data are represented as means ± standard deviations. In the Caco‑2 model, a maximum TEER of 2192±333 Ω∙cm^2^ (1.5 h) and a minimum TEER of 804±240 Ω∙cm^2^ (34.2 h) were observed, whereas in the PBCEC model, the maximum TEER was 813±151 Ω∙cm^2^ (2.5 h) and minimum TEER was 462±148 Ω∙cm^2^ (48 h). TEER data before compound incubation (0 h) reflected the baseline of resistance.
